# Impacts of Milking and Housing Environment on Milk Microbiota

**DOI:** 10.3390/ani10122339

**Published:** 2020-12-09

**Authors:** Bingyao Du, Lu Meng, Huimin Liu, Nan Zheng, Yangdong Zhang, Xiaodong Guo, Shengguo Zhao, Fadi Li, Jiaqi Wang

**Affiliations:** 1 State Key Laboratory of Grassland Agro-Ecosystems, Key Laboratory of Grassland Livestock Industry Innovation, Ministry of Agriculture and Rural Affairs, College of Pastoral Agriculture Science and Technology, Lanzhou University, Lanzhou 730020, China; duby17@lzu.edu.cn (B.D.); lifd@lzu.edu.cn (F.L.); 2 Key Laboratory of Quality & Safety Control for Milk and Dairy Products of Ministry of Agriculture and Rural Affairs, Institute of Animal Sciences, Chinese Academy of Agricultural Sciences, Beijing 100193, China; marilyn0307@163.com (L.M.); liuhuiming521@163.com (H.L.); zhengnan_1980@126.com (N.Z.); yangdongzhang1982@163.com (Y.Z.); guoxiaodong233@163.com (X.G.); Zhaoshengguo1984@163.com (S.Z.); 3 Laboratory of Quality and Safety Risk Assessment for Dairy Products of Ministry of Agriculture and Rural Affairs, Institute of Animal Sciences, Chinese Academy of Agricultural Sciences, Beijing 100193, China

**Keywords:** milk, farming environments, microbiota, 16S rRNA gene sequencing

## Abstract

**Simple Summary:**

Microbiota can enter and persist in raw milk at several stages of the farming chain. The development of culture-independent methods and high-throughput DNA sequencing techniques have improved the approach to study microbiota communities in milk and milk products. This work aimed to determine the effects of farming environments on milk microbiota using the high-throughput DNA sequencing technique, and to elucidate the relationship among the microbiota. The effects of the farming environment on the microbiota in milk can guide farm and management practices to ensure that the milk is not contaminated with bacteria during milking and processing, thereby improving the quality of the milk.

**Abstract:**

The aim of the present study was to determine the effects of farming environments on microbiota in raw milk and to assess the relationship among microbes by 16S rRNA sequencing methods. Samples of raw milk, cow trough water, teat dip cup, teat, teat liner, dairy hall air, cowshed air, feces, feed, and bedding from two farms were collected. The two highest abundant bacterial groups of Moraxellaceae and Staphylococcaceae were found in milk and teat liner samples, respectively, at Zhengzhou farm, Henan Province. Moreover, the two highest abundant bacterial groups of Enterobacteriaceae and Moraxellaceae were found in milk and teat dip cup samples, respectively, at Qiqihar farm, Heilongjiang Province. Source Tracker analysis revealed that the teat liner and teat dip cup were the most important contributors of microbes in milk samples at Zhengzhou farm and Qiqihar farm, respectively, which could be attributed to the management level of the farm. Therefore, disinfection and cleaning procedures should be developed to improve the quality of raw milk.

## 1. Introduction

Milk is a nutritious food as it contains many essential nutrients and biologically active compounds such as lipids, proteins, polyunsaturated fatty acids, fat globule membranes and immunoglobulins [1,2,3,4]. However, milk is susceptible to deterioration and its quality is affected by microbiota contamination. Microbiota spoilage is a contributor to milk product waste and constitutes a potential food safety challenge to human health. The ingestion of milk containing Shiga toxin-producing *Escherichia coli*, *Campylobacter* spp., *Listeria* spp. and *Salmonella* spp. may cause mild to severe illnesses [5]. Microorganisms may contaminate raw milk during milking, transportation, storage and processing, which pose a serious threat to the quality and safety of milk and milk products [6,7,8,9,10].

Culture-dependent and culture-independent methods to analyze microbiota composition have been extensively applied in previous research in raw milk and milk products [11,12,13]. Microorganisms can be isolated and cultured under specific laboratory conditions to improve researchers’ understanding about how they survive, what metabolites they produce, and how they interact with other microorganisms [14]. Vacheyrou et al. adopted a culture-based approach to study possible pathways of microbiota transmission within farms and demonstrated that the type of barns used to house cattle and teat surfaces were the main factors of milk contamination [11]. Quintana et al. adopted a MALDI-TOF approach to study possible contamination routes of on sheep’s milk microbiota on a farm and demonstrated that the farming practices was the main factor for milk contamination [15]. Moreover, the development of culture-independent methods and high-throughput DNA sequencing techniques have improved the approach to study microbiota communities in milk and milk products [10,16,17,18,19,20,21]. For instance, the high-throughput DNA sequencing technique can discover more viable but non-cultivable microorganisms, extremely rare microorganisms in the community can now also be detected, and it can more accurately determine the relative abundance of various microorganisms in the environment.

The effects of the farming environment on the presence of microbiota in milk can guide dairy farms and management practices to prevent milk contamination with bacteria during milking and processing, thus improving the quality of the milk. Additionally, few studies on the microbiota diversity of a farm environment via 16s rRNA sequencing have been reported in China, and specifically, no investigation has been carried out examining the microbiota signatures of different farms for milk in China. Therefore, this work aimed to improve the understanding of the source of microbiota contamination of raw milk by culture-independent methods, and to provide recommendations for the management and prevention of pastures.

## 2. Materials and Methods

### 2.1. Treatment and Sample Collection

Samples at 10 critical control points include raw milk, cow trough water, teat dip cup, teat, teat liner, dairy hall air, cowshed air, feces, feed and bedding were collected from Farm Z in Shijing Village, Guandu Town, Zhongmu County, Zhengzhou City, Henan province (May 2019, temperature 25 °C, herd size = 3200 cows, *n* = 4 (*n* represents the number of samples at each critical control point)) and Farm Q in Fuyu Village, Gannan Town, Gannan County, Qiqihar City, Heilongjiang province (August 2019, temperature = 21 °C, herd size = 10,000 cows, *n* = 3), respectively. All swabs were dipped in sterile NaCl solution (0.09%). For teat samples, the cows’ teats were swabbed with one sterile cotton swab for each teat, which was resuspended in 100 mL NaCl solution in a 225 mL sterile bottle. For teat liner samples, all teat liners of the milking machines were swabbed with one sterile cotton swab for each teat liner and then resuspended in 100 mL NaCl solution in a 225 mL sterile bottle. All sterile cotton swabs were placed in the same solution. For teat dip cup samples, all teat dip cups were swabbed with one sterile cotton swab and then resuspended in 100 mL NaCl solution in a 225 mL sterile bottle. For water samples from the drinking trough, the same volume (approximately 50 mL) of water from the drinking trough of each cowshed was collected and mixed well. To obtain fecal samples, sterile forceps and disposable sterile sampling spoons were used to collect an equal amount of feces (approximately 20 g) from the feces pond of each cowshed, mixed well, and stored in a sterile bag. To obtain bedding and silage samples, sterile forceps and disposable sterile sampling spoons were used to collect an equal amount of samples from each cowshed (approximately 20 g), mixed well, and stored in a sterile bag. Air samples were collected by sedimentation, eluted with physiological saline, and stored in sterile sampling bottles. Then, all farm environmental samples and milk samples were stored at −20 °C prior to DNA extraction.

### 2.2. DNA Extraction

Genomic DNA of microorganisms from milk, water, teat, teat liner, teat dip cup, air, feed, feces and bedding samples were extracted with the HiPure Stool DNA Kits (Magen, Guangzhou, China). For feed, feces and bedding samples, 10 g samples and 90 mL of sterilized saline were vigorously mixed, and the gauze-filtered extracts were centrifuged to collect the pellet for DNA extraction. For milk, water, teat, teat liner, teat dip cup and air samples, a 10 mL sample was centrifuged (14,000× *g* for 5 min) to collect the pellet for DNA extraction.

### 2.3. 16S rRNA Genes Sequencing

The V3–V4 variable region of the 16S ribosomal RNA (rRNA) gene was amplified using primers F341 (CCTACGGGNGGCWGCAG) and R806 (GGACTACHVGGGTATCTAAT). The PCR amplification was achieved with KOD Polymerase (Toyobo, Osaka, Japan) at 94 °C (2 min), followed by 30 cycles at 98 °C (10 s), 62 °C (30 s), and 68 °C (30 s), and ended with a final extension step at 68 °C for 5 min.

FASTP files were analyzed by QIIME version 1.9.1 [22,23]. Paired-end clean reads were joined by FLASH with a minimum 10 bp overlap and mismatch error rate of 2% [24]. Chimera checking and removal of clean tags were achieved using the reference database (version r20110519, http://drive5.com/uchime/uchime_download.html) [25] to perform reference-based chimera checking with the UCHIME algorithm. All chimeric tags were removed, and the effective tags were ultimately obtained for further study. Denoising, chimera detection, and clustering into OTUs (97% identity) were conducted by the UPARSE (version 9.2.64) pipeline [26]. The representative sequences were categorized into organisms via a naive Bayesian model with an RDP classifier (version 2.2) [27] on the basis of the SILVA database (version 132) [28], with the confidence threshold values ranging from 0.8 to 1. Alpha diversity indices were generated in QIIME (version 1.9.1) [22]. Beta diversity results were obtained in R Studio, with Phyloseq and Bray–Curtis distances [29]. Principal coordinate analysis (PCoA) and canonical analysis of principal coordinates (CAP) plots were visualized using ggplot2 [30,31]. The Source Tracker algorithm was utilized to explore potential sources of microbiota contamination in milk samples [32].

## 3. Results

### 3.1. Alpha and Beta Diversities of Microbiota from Raw Milk and Environmental Samples

**Figure 1:**
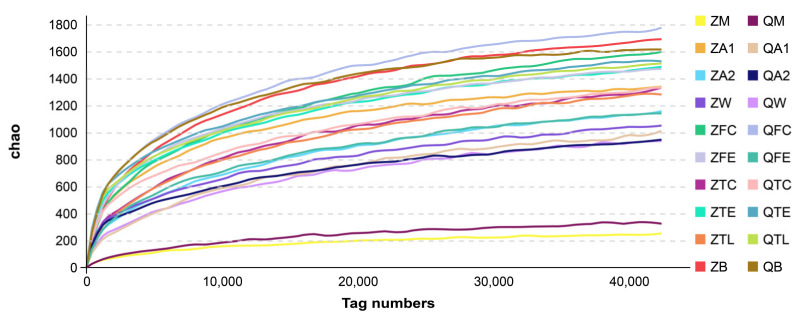
High-throughput sequencing quality control and bacteria alpha diversity indices. ZM: milk samples at Zhengzhou farm, QM: milk samples at Qiqihar farm, ZA1: dairy hall air samples at Zhengzhou farm, QA1: dairy hall air samples at Qiqihar farm, ZA2: cowshed air samples at Zhengzhou farm, QA2: cowshed air samples at Qiqihar farm, ZW: cow trough water samples at Zhengzhou farm; QW: cow trough water samples at Qiqihar farm, ZFC: feces samples at Zhengzhou farm, QFC: feces samples at Qiqihar farm, ZFE: feed samples at Zhengzhou farm, QFE: feed samples at Qiqihar farm, ZTC: teat dip cup samples at Zhengzhou farm, QTC: teat dip cup samples at Qiqihar farm, ZTE: teat samples at Zhengzhou farm, QTE: teat samples at Qiqihar farm, ZTL: teat liner samples at Zhengzhou farm, QTL: teat liner samples at Qiqihar farm, ZB: bedding samples at Zhengzhou farm, QB: bedding samples at Qiqihar farm.

Samples were defined as a possible “source” of microorganisms or a “sink” (a sample that is liable to contain bacteria originating from a source). Sources include all farm environmental samples, and sinks include milk samples [17]. After sequencing, the alpha diversities, beta diversities, principal coordinate analysis and canonical analysis for the bacterial populations were examined. Alpha diversity is a measurement of species richness and evenness in each sample. Quality control, diversity, and richness estimations for each group are shown in Figure 1. The diversity indices of raw milk were low, further confirming the presence of a less-diverse microflora.

### 3.2. Bacterial Group Analysis of Raw Milk, Cow Trough Water, Teat Dip Cup, Teat, Teat Liner, Dairy Hall Air, Cowshed Air, Feces, Feed and Bedding Samples

**Figure 2:**
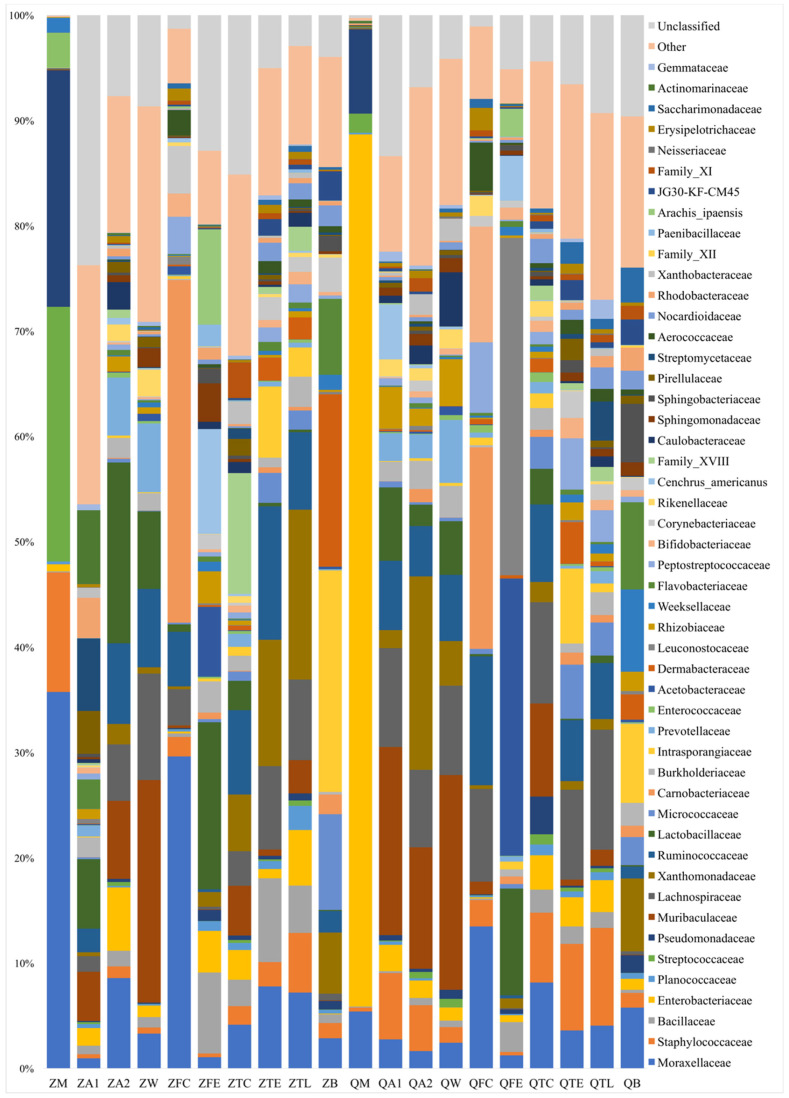
Relative abundance of bacterial families of milk and environmental samples.

For milk samples, the taxa that accounted for >5% in the microbiota included Moraxellaceae (35.7%), Streptococcaceae (24.1%), Pseudomonadaceae (22.4%), and Staphylococcaceae (11.3%) at Zhengzhou farm, and Enterobacteriaceae (82.8%), Pseudomonadaceae (7.9%) and Moraxellaceae (5.4%) at Qiqihar farm. It was noted that the percentage of the taxa Moraxellaceae at Zhengzhou farm samples (35.7%) were much higher than that at Qiqihar farm (5.4%). The relative quantity of Enterobacteriaceae taxa at Qiqihar farm (82.8%) was much higher than that at Zhengzhou farm (0.6%, Figure 2).

The taxa with >5% in the dairy hall air microbiota were different from those in milk microbiota. Actinomarinaceae (6.8%), Streptomycetaceae (6.7%) and Lactobacillaceae (6.4%) were detected from dairy hall air samples at Zhengzhou farm. Muribaculaceae (17.4%), Lachnospiraceae (9.1%), Lactobacillaceae (6.8%), Ruminococcaceae (6.4%) and Staphylococcaceae (6.1%) were detected among dairy hall air samples at Qiqihar farm.

The taxa that accounted for >5% in the cowshed air microbiota were also different from those in milk microbiota. For cowshed air samples, both Lactobacillaceae (17.0%) and Xanthomonadaceae (18.3%) were most commonly found at Zhengzhou farm and Qiqihar farm. The taxa of Moraxellaceae in milk samples from Zhengzhou farm (35.7%) were higher than that in cowshed air samples at Zhengzhou farm (8.52%). Other bacterial groups, such as Ruminococcaceae (7.6%), Muribaculaceae (7.3%), Enterobacteriaceae (5.9%), Prevotellaceae (5.4%) and Lachnospiraceae (5.3%) from cowshed air samples at Zhengzhou farm, and Muribaculaceae (11.5%) and Lachnospiraceae (7.3%) in cowshed air samples at Qiqihar farm, were also identified.

For cow trough water samples, the taxa of Muribaculaceae was the highest at Zhengzhou farm (21.0%) and Qiqihar farm (20.1%), respectively. Other bacteria identified included Lachnospiraceae (10.0%), Ruminococcaceae (7.4%), Lactobacillaceae (7.2%) and Prevotellaceae (6.4%) in cow trough water samples at Zhengzhou farm, and Lachnospiraceae (8.3%), Ruminococcaceae (6.2%), Prevotellaceae (5.9%), Lactobacillaceae (5.0%) and Caulobacteraceae (5.0%) in cow trough water samples at Qiqihar farm.

For feces samples, the taxa Carnobacteriaceae was the highest at Zhengzhou farm (32.4%) and Qiqihar farm (19.0%), respectively. Moraxellaceae (29.5%) and Ruminococcaceae (5.1%) were identified at Zhengzhou farm, and Moraxellaceae (13.4%), Ruminococcaceae (12.2%), Bifidobacteriaceae (10.9%), Lachnospiraceae (8.7%) and Peptostreptococcaceae (6.6%) at Qiqihar farm.

For feed samples, Lactobacillaceae (14.1%) and Leuconostocaceae (31.3%) were the most abundant taxa at Zhengzhou farm and Qiqihar farm, respectively. Bacillaceae (5.9%) and Acetobacteraceae (5.9%) were identified in the feed samples at Zhengzhou farm, and Acetobacteraceae (25.7%) and Lactobacillaceae (9.9%) in feed samples at Qiqihar farm.

For teat dip cup samples, Family XVIII (11.3%) and Lachnospiraceae (9.4%) were the most abundant taxa at Zhengzhou farm and Qiqihar farm, respectively. Other bacteria included Ruminococcaceae (7.9%) and Xanthomonadacea (5.3%) from the teat dip cup samples at Zhengzhou farm, and Muribaculaceae (8.7%), Moraxellaceae (8.0%), Ruminococcaceae (7.2%) and Staphylococcaceae (6.5%) from the teat dip cup samples at Qiqihar farm.

For teat samples, Ruminococcaceae (12.6%) and Lachnospiraceae (8.2%) were the most abundant taxa at Zhengzhou farm and Qiqihar farm, respectively. Other bacteria identified included Xanthomonadaceae (11.9%), Bacillaceae (7.9%), Lachnospiraceae (7.8%), Moraxellaceae (7.7%) and Intrasporangiaceae (6.7%) in the teat samples at Zhengzhou farm, and Staphylococcaceae (7.9%), Intrasporangiaceae (6.8%) and Ruminococcaceae (5.5%) in the teat samples at Qiqihar farm.

For teat liner samples, Xanthomonadaceae (16.0%) and Lachnospiraceae (11.2%) were the most abundant taxa at Zhengzhou farm and Qiqihar farm, respectively. Other bacteria identified were Lachnospiraceae (7.6%), Ruminococcaceae (7.3%), Moraxellaceae (7.1%), Staphylococcaceae (5.6%) and Enterobacteriaceae (5.2%) in the teat liner samples at Zhengzhou farm, and Staphylococcaceae (9.1%) and Ruminococcaceae (5.2%) in the teat liner samples at Qiqihar farm.

For bedding samples, Intrasporangiaceae (20.6%) and Flavobacteriaceae (7.5%) were the most abundant taxa at Zhengzhou farm and Qiqihar farm, respectively. Other bacteria identified included Dermabacteraceae (16.0%), Micrococcaceae (8.9%), Flavobacteriaceae (7.0%) and Xanthomonadaceae (5.7%) in the bedding samples at Zhengzhou farm, and Weeksellaceae (7.1%), Intrasporangiaceae (6.8%), Xanthomonadaceae (6.2%), Trueperaceae (6.1%), Moraxellaceae (5.2%) and Sphingobacteriaceae (5.0%) in bedding samples at Qiqihar farm.

### 3.3. Principal Coordinates Analysis (PCoA) Shows Relationships between Raw Milk and Environmental Samples

**Figure 3:**
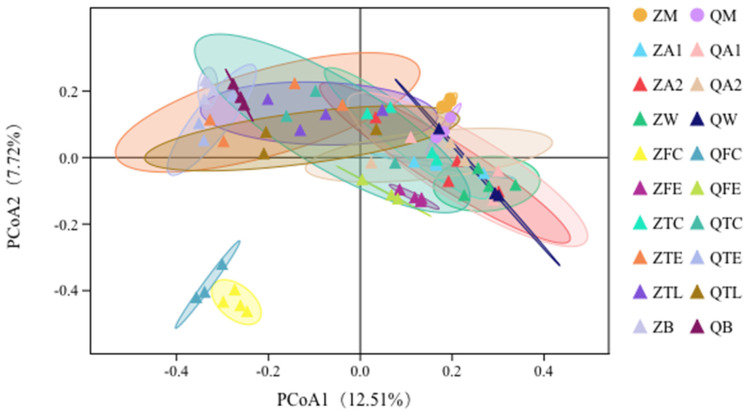
Principal coordinates analysis (PCoA) plot of milk and environmental samples.

We further explored the relationship of taxa versus milk, water, teat, teat liner, teat dip cup, air, feed, feces, or bedding samples through principal coordinates analysis (PCoA). In this plot, the samples forming clusters were observed (Figure 3). There are several broad similarities between samples collected from the same habitat. Within both habitats, it was apparent that the data points representing the milk sample microbiota overlapped with farm environmental samples, reflecting similarities in their beta diversities. The fecal samples from both habitats were separated from the milk samples and were different from the corresponding samples from the same environment (Figure 3) while the milk samples were relatively closely related to other environmental samples.

### 3.4. Canonical Analysis of Principal Coordinates (CAP) Shows Relationships between Raw Milk and Environmental Samples

**Figure 4:**
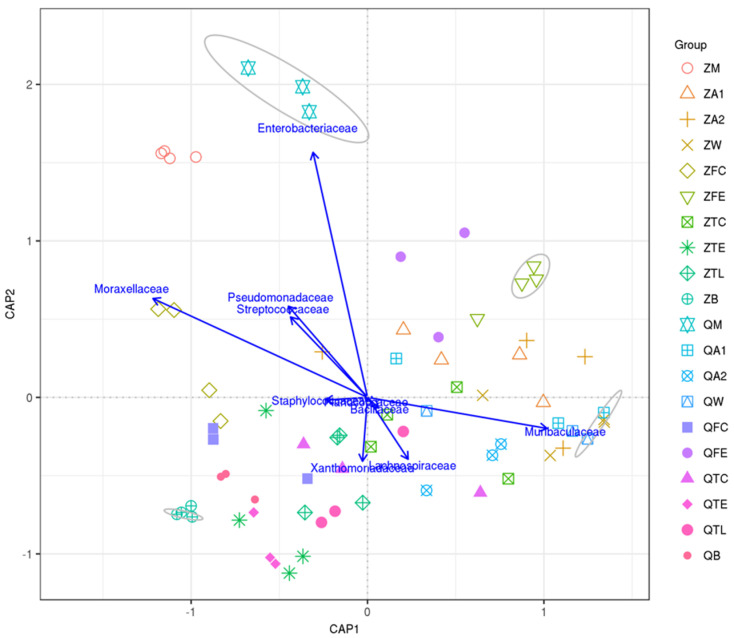
Canonical analysis of principal coordinates (CAP) illustrated the correlations of the 10 major OTUs within different samples. Samples enclosed in a gray circle are considered to be in the same group at a 70% similarity level.

Canonical analysis of principal coordinates (CAP) demonstrated the top taxa (based on the top 10 most abundant OTUs) associated with milk, cow trough water, teat dip cup, teat, teat liner, dairy hall air, cowshed air, feces, feed and bedding samples (Figure 4). Milk samples in Qiqihar, prominently characterized by Enterobacteriaceae, were considered as independent groups at a 70% similarity level. However, one group was found in the water samples at Zhengzhou farm and Qiqihar farm by a high relative abundance of Muribaculaceae.

### 3.5. Source Tracker Model Further Evaluates the Contribution of Environmental Sample Sources to the Raw Milk Microbiota

**Figure 5:**
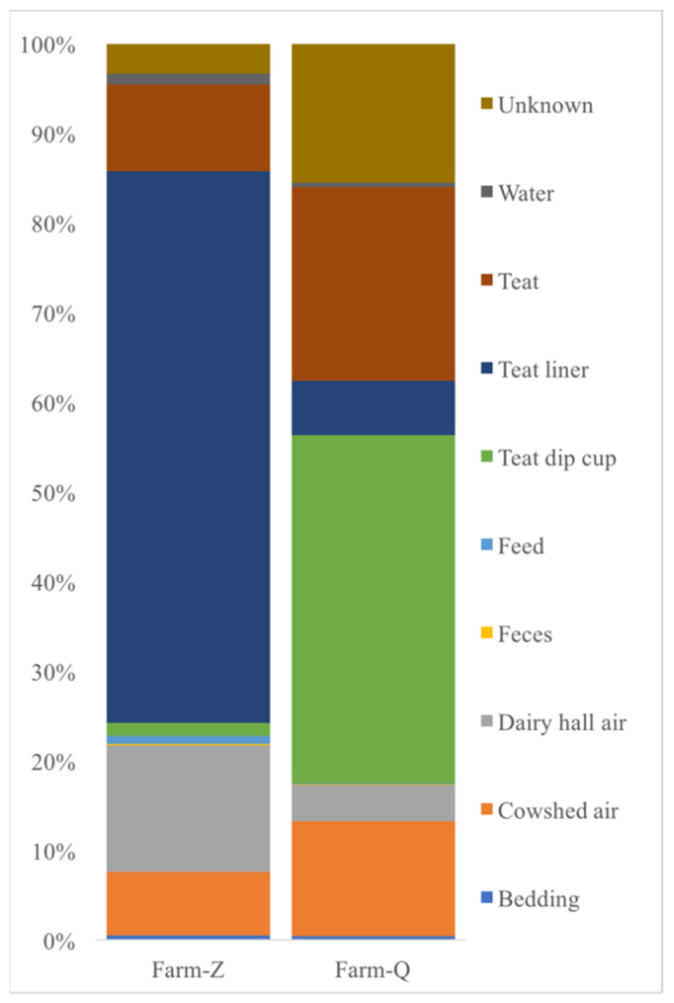
Source Tracker results highlight the percentages of inferred sources of contamination in milk samples. Farm-Z: Zhengzhou farm, Farm-Q: Qiqihar farm.

The Source Tracker model considered each individual community (such as milk, cow trough water, teat dip cup, teat, teat liner, dairy hall air, cowshed air, feces, feed and bedding samples) as a mixed community deposited from other known or unknown environmental sources (Figure 5).

The study considered that the teat liner had a significant correlation with microbes found in farm milk samples at Zhengzhou farm, and the teat dip cup had a significant correlation with microbes recovered from farm milk samples at Qiqihar farm. Dairy hall air and teat were the next most important sources of contaminants in Zhengzhou farm and Qiqihar farm milk samples, respectively.

## 4. Discussion

In the milk microbiota, the relative abundances of Moraxellaceae and Staphylococcaceae were high at Zhengzhou farm. Although different relative abundances were observed between milk and teat liner samples, Moraxellaceae and Staphylococcaceae were found to be the most abundant taxa in milk and teat liner samples, respectively. Furthermore, the Source Tracker analysis indicated that the microbes identified from the teat liner had a significant correlation with the microbes identified in milk samples at Zhengzhou farm. The results were consistent with a previous study of psychrotrophic bacteria from teat liners, where liner disinfection did not result in a significant reduction in the number of target bacteria [33]. The presence of contamination suggests a possible scenario that the sanitizing procedures are not effective in neutralizing microbiota or there might be a secondary contamination. In addition, if the water used is under 60 °C, the sanitizing procedures are not effective. It is important to check the water temperature after the cleaning cycle. All in all, the presence of microbiota in teat liners represents a challenge.

The relative abundances of Moraxellaceae were high in milk and feces at Zhengzhou farm; thus, the high proportion of Moraxellaceae in milk might suggest that bacteria were transferred from feces to milk. Therefore, farm managers should formulate reasonable cattle farm manure cleaning measures and cow udder cleaning measures to prevent feces from remaining on the udders and contaminating milk.

High proportions of Streptococcaceae and Pseudomonadaceae were found in milk samples at Zhengzhou farm. However, the relative abundances of Streptococcaceae and Pseudomonadaceae were low in cow trough water, teat dip cup, teat, teat liner, dairy hall air, cowshed air, feces, feed, and bedding samples at Zhengzhou farm. This suggests that these microorganisms may remain in areas with dairy bulk tanks or milk pipelines that were difficult to disinfect and eradicate. These results were similar to those described by Zou et al. [34], where microbiota isolates were detected from the interior surfaces of the facilities and the distribution of strains had high species diversity, indicating that incomplete disinfection or secondary pollution in milk product processing lines. Quintana et al. studied the possible routes of contamination of the milk microbiota of farm sheep and proved that farming methods are the main factor for milk contamination. Statistical models show that the factors that affect the number of microorganisms in milk are related to the frequency of acid treatment when cleaning milking machines [15]. Thus, it suggests that facility management should increase the frequency of equipment cleaning while paying greater attention to its cleanliness.

At Qiqihar farm, Enterobacteriaceae were the most abundant taxa (82.8%) in milk, but their proportion in teat dip cup samples was 3.2%. Moraxellaceae abundance was relatively low (5.4%) in milk samples, but its proportion was 13.4%, 8.0% and 5.2% in feces, teat dip cup and bedding samples, respectively. The Source Tracker analysis from this study indicated that the teat dip cup samples had a significant overlap of microbes found in milk samples at Qiqihar farm. This correlation may easily be overlooked. A previous study demonstrated that ineffectiveness of dipping agents, dirty udders and incomplete teat dip cup cleaning are among the highest risk factors for bacterial contamination in farms [35]. Incomplete cleaning and disinfection of milk liners and teat dipping cups with automatic milking systems in dairy farms resulted in the relatively high prevalence of these pathogens in Galicia [36]. Therefore, regular evaluation of efficacy and replacement of these dipping agents, and regular cleaning of teat dip cup, should be carried out.

## 5. Conclusions

In this study, through analysis and comparison, we demonstrated the differences between the microbiota present in milk from that of cow trough water, teat dip cup, teat, teat liner, dairy hall air, cowshed air, feces, feed and bedding. The Source Tracker analysis revealed that the teat liner and teat dip cup were the most significant contributors to bacterial contamination in milk samples at Zhengzhou farm and Qiqihar farm, respectively, which could be resolved at farm management levels. Therefore, reasonable disinfection and cleaning procedures should be provided by farm managers. Regular evaluation of the efficacy and replacement of these dipping agents, and regular cleaning of teat liners and teat dip cups, should be performed to improve staff awareness of cleanliness and hygiene requirements.
